# Impact Evaluation of a Clinical Point-of-care Ultrasound Intervention in an Academic Emergency Department

**DOI:** 10.5811/westjem.53918

**Published:** 2026-06-28

**Authors:** Alyssa Platt, Timothy Peterson, Erica Peethumnongsin, Maragatha Kuchibhatla, Rebecca G. Theophanous

**Affiliations:** * Duke University School of Medicine, Department of Biostatistics and Bioinformatics, Durham, North Carolina; † Duke University School of Medicine, Department of Emergency Medicine, Durham, North Carolina

## Abstract

**Introduction:**

Point-of-care ultrasound (POCUS) can expedite patient diagnoses and improve procedural safety in emergency department (ED) patients. Nevertheless, clinical POCUS use lags in many ED sites nationwide, with only 40% of community physicians using POCUS according to a study from 2019–2021. We studied the impact of a multifaceted clinical POCUS intervention to address barriers and increase clinical POCUS use.

**Methods:**

We conducted a prospective cohort study at a single academic hospital that included ED attendings, residents, and advance practice clinicians (APC). A multifaceted four-week intervention for emergency clinicians from December 2023–January 2024 addressed barriers to POCUS use identified on a pre- and post-intervention survey. The intervention included POCUS education during clinical shifts by ultrasound faculty, clinical POCUS workflow demonstration during resident conference/faculty meetings, and QR code reference files on machines for a total of two hours of additional training per clinician. The primary outcome was the number of clinical ED POCUS performed during the 18 months pre-intervention and 12 months post-intervention. Secondary outcomes were POCUS workflow knowledge exam scores and POCUS revenue. We analyzed clinical POCUS numbers from July 2022–December 2024 using an interrupted time series model.

**Results:**

Forty-two of 99 emergency clinicians (42.4%) responded to surveys pre-intervention and 28 post-intervention (28.3%). Fifty-six physicians/APCs (56.6%) participated in the in-person POCUS intervention (17 attendings, 34 residents, 5 APCs). The unadjusted number of POCUS exams performed increased from 6,708 pre-intervention (18 months) to 11,600 post-intervention (12 months). After controlling for the underlying secular trend, we saw no effect of the intervention with no significant increase in overall number of POCUS exams performed per 100 ED patients after the intervention (mean level change = −0.81, 95% CI, −2.43 to 0.82; *P* = .33). Neither was there an increase in the slope of the secular trend post-intervention (difference in slope, β = 0.00, 95% CI, −0.10 to 0.10; *P* = .98 for post-intervention, β = 0.02, 95% CI, −0.08 to 0.12 for sustainability period). Monthly POCUS revenue increased ~$55,000 in total billed and $10,000 reimbursed (~16% reimbursement). This was the professional component only that was billed by the physician and not the technical component covering equipment and maintenance. Post-intervention mean knowledge scores were 88% correct (standard deviation 12.0) with 16/22 participants (72.7%) passing the assessment (score > 90%).

**Conclusion:**

A multifaceted intervention was not shown to improve clinical point-of-care ultrasound use and revenue when accounting for an underlying sustained secular upward trend in number of POCUS exams performed per 100 emergency department patients throughout the study period. Future interventions directed toward low POCUS users and identification of clinical POCUS champions may improve clinical POCUS use.

## INTRODUCTION

Use of point-of-care ultrasound (POCUS) can improve patient care by expediting diagnosis in both medical and trauma emergency department (ED) patients.^1^,^2^ Studies show that POCUS use improves first-pass success in central line placement and other procedures and decreases complication rates.^1^,^2^ Emergency medicine (EM) guidelines recommend POCUS as a bedside clinical tool to improve patient care.^3^,^4^ Emergency ultrasound streamlines patient care workflows by reducing radiology orders and transport of critically ill patients, yielding improved ED and hospital throughput and cost efficiencies.^1^,^5^ Performing POCUS also improves patient satisfaction with direct hands-on interaction at the bedside.^1^,^5^

The American College of Emergency Physicians and the Accreditation Council for Graduate Medical Education guidelines require POCUS training and performance as a core diagnostic and procedural skill for residency graduation.^3^,^4^ Nevertheless, < 50% of faculty at most clinical sites in the United States are credentialed to teach and perform POCUS.^1^,^6^,^7^ Although EM is a leading specialty for POCUS education and training, many emergency physicians who trained prior to 2008 lack the necessary training and skills to clinically perform POCUS, creating a persistent gap between POCUS training and clinical use.^1^,^6^–^9^ Furthermore, Russell and Smalley et al found that community emergency physicians performed POCUS on only 40% of patients from 2019–2021 compared to 68% of academic physicians, despite adequate ultrasound equipment.^10^,^11^ Studies at the Veterans Affairs (VA) healthcare system and rural EDs have identified several important factors contributing to this gap in POCUS use. The most common barriers are lack of POCUS training (84% of VA sites in 2020), lack of a systemwide POCUS documentation and archiving program (35%), and lack of hospital resources or funding (28% lack funding for support staff and 50% for training). Fewer sites reported a lack of adequate POCUS equipment (29%).^8^,^9^,^12^–^17^

Multiple studies have sought ways to increase clinical POCUS uptake and use, but no standardized process exists. Mosier et al identified expert ultrasound faculty, simulation training, and regular review sessions as necessary to improve ultrasound training and use.^2^ Furthermore, Amini et al reported that 75% of the 70 nonacademic EDs included in their study did not have someone responsible for reviewing POCUS images for quality assurance (QA), and 50% of sites did not have a mechanism for archiving images, which are both areas for potential POCUS system improvements. Some studies have implemented an institution-specific POCUS program for EDs or inpatient services with variable success.^18^–^22^ Thus, additional studies are needed to address the barriers identified throughout the clinical POCUS pathway for a comprehensive program including image acquisition, documentation, archival, quality review, and billing processes.^18^–^22^

**Population Health Research Capsule**

What do we already know about this issue?

*Point-of-care ultrasound (POCUS) can expedite diagnoses and improve procedural safety, yet clinical use lags in hospitals nationwide*.

What was the research question?

*We studied the impact of a multifaceted clinical POCUS intervention to increase clinical POCUS use*.

What was the major finding of the study?

*A program to enhance use of POCUS was not effective at increasing ultrasound scans performed per 100 patients (mean change = −0.81, 95% CI, −2.43 to 0.82; P = .33), or increasing professional revenue over general trends (+ 16%)*.

How does this improve population health?

*Future interventions directed toward identification of clinical POCUS champions may improve clinical POCUS use*.

### Objective

Our study objective was to evaluate the impact of a clinical POCUS intervention built using barriers and facilitators to POCUS use, which were identified by emergency physicians and advanced practice clinicians (APC) at a single academic hospital. Our goal was to increase the number of ED-performed clinical POCUS exams and revenue post-intervention, developing a model for future dissemination to other EDs and healthcare sites.

## METHODS

### Study Design

We performed a prospective cohort study from July 2022–December 2024 that identified barriers and facilitators to clinical POCUS use by EM attending and resident physicians and APCs working at a single academic hospital ED, which is described in a separate paper.^23^ To complement the prospective cohort study, we also conducted a retrospective analysis of electronic health data extracted from the ED POCUS Butterfly^®^ archiving system beginning July 1, 2022 and including a sustainability period through December 31, 2024. The study was performed according to the Strengthening the Reporting of Observational Studies in Epidemiology (STROBE) guidelines^24^ (Supplemental File 1), The study was approved by the institutional review board (Pro00114618) and followed ethics guidelines.

### Study Setting

We conducted this study at a single, tertiary-care, academic, Level I trauma center with 1,048 inpatient hospital beds, almost 85,000 annual ED visits, and 29.8% admissions (ED patients admitted to the hospital). The ED’s diagnostic ultrasound machines included two General Electric Healthcare Venue cart-based systems, three Sonosite M-Turbos, and two Sonosite Edges (with two new Sonosite X-porte machines added in July 2024). The ED also had three procedural vascular Sonosite Nanomaxx machines. The four Butterfly^®^ IQ handheld ultrasounds with clinical capability were only used during educational sessions. The study site had an emergency ultrasound director and five emergency ultrasound-fellowship trained faculty. The EM program had 12 residents per academic year and 1–2 emergency ultrasound fellows. Epic Maestro Care was the electronic health record (EHR), and Butterfly^®^ Network cloud was the POCUS manager for image storage and archival. Ultrasound faculty performed QA image review weekly for all clinical POCUS exams.

### Selection of Participants

All clinicians working in the ED during the study period were invited to participate. The target study population included 45 attending physicians, 19 APCs, and 35 residents (postgraduate years [PGY] 1–3). All emergency physicians and APCs were invited to participate in both the surveys and training sessions with participation advertised via email, at weekly EM resident conference, and at monthly EM faculty meetings.

### Data Collection

All ED attendings, residents, and APCs were emailed a secured REDCap survey (with a website link and QR code) pre- and post-intervention to assess their understanding of the ED clinical POCUS workflow and their opinions on barriers or potential facilitators for the process. The survey was created by ultrasound-content experts and based on validated surveys from other POCUS studies.^18^,^19^,^21^,^25^ Responses were based on a five-point Likert scale.

The clinical POCUS intervention is described in a prior paper. Briefly, we identified POCUS use barriers and facilitators at our facility with a pre-survey one month pre-intervention (November 2023). Then, over a four-week period emergency physicians and APCs underwent a multifaceted training intervention to address these barriers. This training involved the following: 1) emailing an online presentation and creating QR codes with a reference document outlining the standardized POCUS image acquisition and archival process; 2) presenting the current clinical POCUS workflow process at an ED faculty meeting, faculty development session, and EM resident conference day; and 3) ultrasound faculty leading an in-person demonstration of the clinical POCUS process for small groups of emergency clinicians during clinical shifts. We invited emergency physicians and APCs to participate in a six-month post-intervention survey including a knowledge exam to obtain feedback on the POCUS intervention and assess workflow improvements (Supplemental File 2).

Ultrasound faculty extracted data from the Butterfly^®^ ultrasound clinical archiving system on the number and type of POCUS exams done in the ED; subsequently, authorized administrative support staff in the ED. extracted clinical POCUS billing data for the study team. Daily ED patient volume was obtained from ED administrative leadership.

### Study Outcomes

Our primary outcome measure assessing the impact of the POCUS program intervention was the number of clinical POCUS exams performed by emergency physicians and APCs (per 100 patients) during three distinct periods including pre-intervention (July 1, 2022–December 18, 2023), post-intervention (December 19, 2023–June 30, 2024), and a sustainability period (July 1–December 31, 2024) as measured by counts of POCUS exams saved to Butterfly^®^ software. Additional outcomes included the type of clinical POCUS exams being performed, ED POCUS billing and reimbursement data, self-perceived POCUS frequency, and a knowledge assessment on clinical POCUS workflow. We hypothesized that clinical ED POCUS use would increase post-intervention.

### Data Sources

We used Epic Maestro Care to obtain the daily number of ED patient visits and Butterfly^®^ Network software to review POCUS exams performed in the ED. Surveys built using the institution’s secured REDCap server were emailed to emergency clinicians pre-intervention in November 2023 and six months post-intervention in June-August 2024, with reminders sent weekly for two weeks. We retrospectively obtained ED clinical POCUS billing data from administrative and support staff six months post-intervention.

### Bias

Our principal investigator (RGT) wrote the survey, and to reduce bias it was edited by ED ultrasound faculty experts who were not study team members. The survey was modeled after ultrasound surveys used and tested in other published studies.^16^–^18^,^25^ The survey was pilot tested with an emergency physician at another local institution with research training who was not eligible for the study.

Participants were enrolled with implied consent after reading the study information sheet with the REDCap survey link emailed by the PI. All participation was voluntary with participant withdrawal allowed at any time. Only study personnel had access to data, which was secured in an institutional database as per standard research protocols. Participant names and dates linked pre-/post-intervention survey data to assess for improvement after the POCUS intervention and were necessary for analysis of responses and to maximize process improvements. Survey data was deidentified by the study PI and coded with a key for biostatistician review and analysis. All POCUS studies are saved within the institution’s approved and secured Butterfly^®^ cloud system and written reports in Epic Maestro Care as part of standard clinical care. Deidentified data was presented to the ED group for feedback and process improvements.

We evaluated our POCUS intervention using Kirkpatrick’s levels of learning, assessing participants’ reaction to the POCUS intervention through REDCAP surveys and their learning through an 11-question multiple-choice knowledge assessment on clinical POCUS workflow. We evaluated behavior via in-person observed demonstrations of faculty completing a clinical POCUS exam during on-shift teaching. Finally, we reviewed ED POCUS program costs and revenue.^26^

### Statistical Methods

We summarized patient characteristics and descriptive outcomes using means (with standard deviations) and medians (within interquartile range) for continuous variables and counts with percentages for categorical variables. Survey data collected both pre- and post-intervention were summarized by time period. We summarized time series analyses descriptively by aggregated time period (ie, weeks for POCUS data or months for billing data).

We analyzed our primary outcome—the number of Butterfly^®^ POCUS exams performed—using interrupted time series models with exams aggregated to weekly counts, divided by total weekly ED patient volume, and scaled to number of exams per week per 100 ED patients. Multiple types of POCUS exams performed during the same saved Butterfly^®^ session were counted as separate exams. We extended the time series back one year and six months prior to the start of the intervention (ie, starting in July 2022) to better estimate a secular trend and control for any seasonal fluctuations in exam rates. Interrupted time series models were implemented using linear regression models with a fixed effect for secular trend (ie, weeks from July 1, 2022), an indicator for post in-person training, and a fixed effect for number of weeks from the end of the training period that took a value of zero before the training period and count of weeks after the training period.

Similarly, we included indicator and week count fixed effects for the sustainability period. We reported the regression constant and indicator for post-training and sustainability periods to quantify rates at the beginning of the study period, post-training, and sustainability periods, respectively. We reported parameter estimates for the secular trend and a linear combination of secular trend and post-intervention trends to quantify slopes pre- and post-intervention. To aid in interpretation of time series analysis, we plotted observed weekly rates (per 100 ED patients) alongside fitted regression lines for pre- and post-training periods. We excluded exam rates between December 19, 2023–January 31, 2024 (ie, the training period) from analysis because the intervention had not been fully implemented and its relatively short duration precluded reliable estimation of a separate time trend. To ascertain whether regressions needed to incorporate autoregressive error structure, we used a Breusch-Godfrey test for higher order serial correlation to test for up to six lags, and we computed the Durbin-Watson *d* statistic to confirm first-order serial correlation (if present).^27^,^28^

Missing data from blank responses on surveys are reported in results tables. Characteristics of respondents were compared by survey response pattern (ie, responded to both pre- and post-surveys versus responding to one or the other) to add context to pre/post results and potential impact of missing data via non-response. We calculated *P* values using an alpha of 0.05 for statistical significance. With only one primary outcome, no adjustments were made for multiple testing. All analyses were performed using Stata v19 software (StataCorp, LLC, College Station, TX).

## RESULTS

### Characteristics of the Study Subjects

In the 18-month pre-intervention to six-month post-intervention plus six-month sustainability period from July 1, 2022–December 31, 2024, a total of 128,468 adult ED patients were seen and 18,308 POCUS exams were performed. Of 103 emergency physicians and APCs, four attendings were on the study team and ineligible for the survey to prevent bias. Forty-two eligible clinicians (42.4%) responded to the pre-intervention survey, including 26 EM attendings (59.1% of eligible), 14 EM residents (38.8% of eligible), one APC (5.3% of eligible), and one unidentified user. The post-intervention survey had 22 analyzable responses (22.2% of eligible) after removing six duplicate responses. Additional participant survey characteristics are described in the prior paper and in Table 1.^23^ Attendance at the in-person training was 60 total (17 attendings, 35 residents, six APCs) among 54 individual participants and 17 sessions, with attendance ranging from 1–12 individuals per session.

Per survey responses, there were 26 male participants (51.0%), 25 female (49.0%), 9 Hispanic/Latin/x/Spanish origin (18.8%), with mean age 35 (SD 7.6) (range 29–38 years of age), and a mean 8.0 years (7.5) of clinical work in EM (range 2.2–10 years). Fourteen participants (26.9%) reported fellowship training. Responses per level of training included the following: three APCs (5.8%); six PGY-1 residents (31.6%); seven PGY-2 (36.8%), six PGY-3 residents (31.6%); no fellows; and 30 attending physicians (57.7%) (Table 1). Characteristics of the 42 pre-intervention and 22 post-intervention survey respondents were largely similar, with more respondents having fellowship training and POCUS training in residency.

#### Objective Use of Point-of-care Ultrasound (Butterfly^®^ Data and Billing Data)

**Figure 1:**
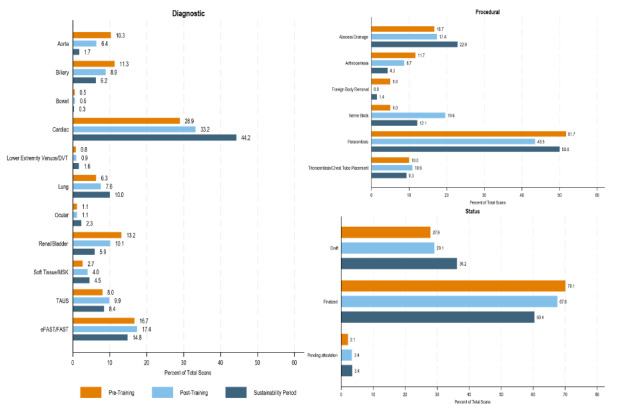
Diagnostic and procedural Butterfly^®^ point-of-care ultrasound exam types and exam status by study period. *We excluded the following diagnostic exam types with low frequencies: one appendix exam in the pre-intervention period, one in the sustainability period, and none in the post-intervention period); 13 obstetrics/gynecology exams in the pre-intervention period, and none in the post-intervention or sustainability periods; and one pediatric abdomen exam in the pre-intervention period and none in the post-intervention or sustainability periods.

Excluding those performed during the training period, there were 18,308 POCUS exams performed from July 1, 2022–June 30, 2024, with 5,720 (31.2%) recorded in “draft” status and 12,066 (65.9%) with “finalized” status (Supplemental Table 1). The most common diagnostic exam type was cardiac (*n* = 4,042 scans, 36.1% of diagnostic exams), while the most common procedural guidance POCUS type was for paracentesis (*n* = 121, 55.3% of procedural guidance exams). There was little difference seen in types of diagnostic or procedural guidance POCUS, except for a modest increase in the proportion of POCUS exams that were cardiac in the sustainability period and a modest decrease in proportion of exams with finalized status in the sustainability period (Figure 1). At our site we do not save images for vascular access; thus, those numbers are not reported under procedures. All clinical POCUS exams (100%) are archived in Butterfly^®^.

Durbin Watson and Breusch-Godfrey tests showed no evidence of autocorrelation of errors; thus, we used a simple ordinary least squares regression with robust standard errors and no additional autoregressive error structure. Rate of POCUS per 100 ED patients at week 0 (ie, July 1–7, 2022) was 3.88 (95% CI, 3.09–4.66), and there was evidence of a sustained secular upward trend in exams per 100 ED patients during the pre-intervention period (pre-intervention slope, β = 0.03, 95% CI, 0.01–0.04; *P* < .001) (Table 2).

**Figure 2:**
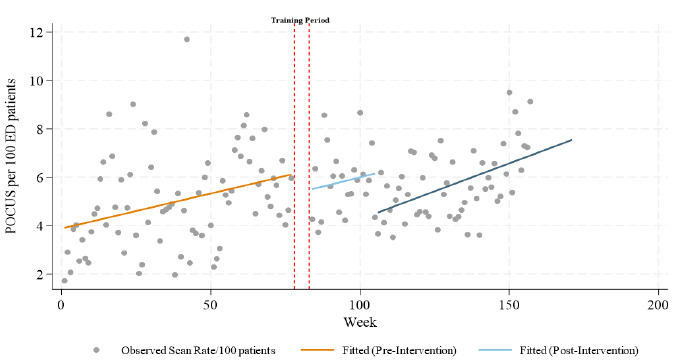
Raw point-of-care ultrasound exam counts (all types) with fitted linear regression lines pre- and post-intervention in a study to assess the impact of a clinical intervention to address barriers and increase clinical point-of-care ultrasound use. *ED*, emergency department; *POCUS*, point-of-care ultrasound.

After controlling for the underlying secular trend, there was no significant increase in overall level of POCUS per 100 ED patients after the intervention (mean level change = −0.81, 95% CI, −2.43 to 0.82; *P* = 0.33) and no increase in the slope of the secular trend post intervention (difference in slope, β = 0.00, 95% CI, −0.10 to 0.10; *P* = .98 for post-intervention; difference in slope, β = 0.02, 95% CI, −0.08 to 0.12 for sustainability period). Level is defined by the overall mean number of POCUS during a particular time frame. Raw and fitted values for the time series are visualized in Figure 2.

Approximately 16.5% of finalized studies were QA reviewed pre-intervention and 22.5% post-intervention. Concomitant with the percentage increase in number of clinically billed POCUS exams, amounts billed and reimbursed for POCUS also increased over the course of the six months prior to. and six months post intervention (Supplemental Figure 2). Overall, post-intervention monthly POCUS revenue increased ~$55,000 in total billed and $10,000 reimbursed (approximately 16% reimbursed). However, the causal linkage between the intervention and financial impact is unclear without interrupted time series modeling.

### Intervention Evaluation and Knowledge Scores

Mean knowledge scores on post-surveys were 88% correct (SD 12.0) with 16 participants (72.7%) passing the assessment (score > 90% or a score of ≥ 10 correct answers) (Table 3).

The most frequently missed knowledge test questions were number of days to complete and sign a study before it disappears (*n* = 11 correct, 50%) and requirements to perform and document an educational POCUS (*n* = 16 correct, 72.7%) (Supplemental Figure 3).

Post-intervention, 17 of 22 (81%) respondents found the POCUS education/training intervention (which included ultrasound faculty live demos on shift, PowerPoint/online presentation, and QR codes to reference material reviewing ultrasound machine use and Butterfly^®^ documentation) to be useful. Eight respondents (36.4%) found the training intervention very useful, six (27.3%) somewhat effective, eight (28.6%) felt neutral about the intervention, and two respondents thought it was somewhat ineffective(Table 3).

## DISCUSSION

Our POCUS intervention did not show a statistically significant change in mean level of POCUS use per 100 patients post-intervention after adjusting for a secular trend with increasing numbers of POCUS exams performed throughout the study period. The ED leadership and faculty development sessions have focused on increasing ED POCUS use over the past 2–3 years, potentially contributing to the positive POCUS use slope leading into the intervention period. Thus, the ongoing longitudinal POCUS teaching at education conferences and faculty development sessions made it difficult to isolate the dependent effect of the discrete POCUS training intervention.

The combined in-person plus online training format was similar to the intervention reported by Schnittke et al, integrating in-person resident education on POCUS documentation and archiving.^18^ However, that study was limited to EM residents, whereas we also included attendings and APCs as regular clinical POCUS users. Additional studies support hands-on learning as the mainstay of POCUS education.^19^,^21^ Russell et al found that 87% of medical students had increased confidence in identifying the gallbladder and performing a cardiac exam and 98% reported increased confidence with performing a focused assessment with sonography in trauma (FAST) exam after learning POCUS through hands-on integration into anatomy, physical examination skills, and clerkship courses.^1^

In contrast to community EDs where POCUS knowledge is identified as a major barrier, our study found high mean knowledge scores (88% correct), with 16 participants passing the assessment (> 90% correct).^6^,^7^,^10^,^11^ Results suggest POCUS workflow knowledge retention at six months post-intervention, although we do not have further sustainability data. Janjigian et al also found POCUS knowledge and skill retention (*knowledge* median score of 85% post-course to 81% at 12 months post-intervention and *hands-on image acquisition skills* median score 75% post-course to 74% at 12 months post-course) in 23 hospitalists at four clinical sites after participating in a two-day hands-on POCUS course plus longitudinal phase curriculum.^21^ The academic hospital environment with continuous resident training, frequent lectures, and hands-on education sessions likely contributed to the increased comfort with POCUS reported by participants.

Nevertheless, a gap remains between POCUS education and clinical POCUS use, which is true at most clinical sites nationwide.^8^,^12^–^14^ When reviewing our clinical POCUS data, we found that almost 30% of clinical POCUS exams remained in draft status (eg, the worksheet was incomplete or the study was unsigned, preventing it from being published in the clinical chart or from being billed). A small percentage of studies were “pending attestation” (about 2% pre-intervention and 3% post-intervention), which means the EM resident or APC saved their POCUS images and completed the exam worksheet, but an attending physician did not sign it. Therefore, the images could not be published in the EHR and be billed. Also, participants reported sometimes performing POCUS without saving images on the machine (eg, “phantom” exams). These workflow problems create an opportunity for individualized feedback focusing on emergency clinicians who are performing fewer POCUS exams and not completing the process. The same problem is shared across POCUS leadership and hospital sites nationwide; thus, additional work is needed to better understand where the breakdown in workflow processes is occurring and how it can be improved.^2^,^12^–^14^,^18^

Regarding intervention impact evaluation, most respondents (17/22) found the POCUS education/training intervention to be useful (eg, in-person plus online reminders of the clinical workflow process). Additional repetitive sessions could further bolster reinforcement and likely increase clinical POCUS uptake. Our prior studies done with clinicians of mixed training backgrounds (internal, family, and emergency medicine) at the VA hospital found strong support for peer influences and feedback as facilitators to POCUS use.^17^ Similar positive reinforcement could be implemented at our ED site. Furthermore, identification of POCUS champions has increased POCUS use in other studies, including at the VA hospital, and could be promoted to remind people about appropriate POCUS use.^17^,^19^,^29^

The most common diagnostic exams performed are cardiac (about 36%), extended € FAST (about 16%), biliary (about 9%), and aorta (about 6%). The number of these exams, particularly cardiac and eFAST, are likely higher than would be seen at a community ED. This is partially because our academic institution’s patients can be seriously ill with recent surgical or invasive procedures, frequent malignancy, organ transplant, or subspecialty population rates, and increasing complexity of transferred patients. Furthermore, our hospital’s status as a Level I trauma center increases the proportion of exams evaluating for trauma such as cardiac, lung, and eFAST, necessitating performance of these POCUS exams.

The most common procedural applications were paracentesis (almost 54%) and abscess drainage (17–21%), which are also common ultrasound-guided procedures being performed at VA and community EDs.^6^,^7^,^14^,^17^ Ultrasound-guided vascular access is also a common indication, but numbers could not be reported because those images are not saved at our site. Therefore, the types of POCUS exams being performed will be guided by the local ED environment to facilitate both expedited diagnosis and safer procedures.

Our recent parallel study at the local VA involved clinicians of mixed training backgrounds and focused only on ED attending physicians plus one APC, identifying a statistically significant increase in clinical POCUS exams from 72 to 267 performed in six months.^17^ The lack of statistical significance for our current study could be because all clinicians were EM-trained here, potentially starting at a higher baseline rate of POCUS knowledge and skills. Furthermore, the clinical environment of EM residents eager to learn and perform procedures may have increased the overall number of POCUS exams. Both studies support standardized ED POCUS documentation and an archiving system, with simple steps to perform the POCUS exam and a clear documentation protocol.^13^,^15^,^17^ Future studies should address time constraints on shift, ease of locating machines with designated storage areas, and repetitive or longitudinal POCUS training opportunities.^30^–^32^

Finally, a comprehensive POCUS system costs money to establish and maintain but can generate revenue for physicians and the healthcare system.^32^,^33^

Estimated costs for a comprehensive POCUS program based on prior clinical and administrative data include 0.25 faculty hours for a POCUS director for 12 months; approximately $15,000 to host a large-group POCUS training course for 20–25 participants; an estimated $100,000 for two cart-based diagnostic machines and one vascular access machine; and between $50,000–$100,000 for an ultrasound-archiving software license, implementation, and five-year maintenance costs.^17^ Although the causal linkage between the intervention and financial impact is unclear without interrupted time series modeling, the numerous POCUS studies still in draft status signifies high potential revenue loss both pre-intervention (1,869/6,708 studies, 27.9%) and post-intervention (727/2,603, 27.9%). Other studies that have performed financial analysis of POCUS systems show significant cost savings and program effectiveness through increased use of POCUS, decreased number of radiology studies, and improved ED length of stay and hospital throughput.^32^,^33^ Thus, establishing and maintaining a clinical POCUS system should be beneficial to healthcare facilities.

## LIMITATIONS

The single academic hospital setting limits study generalizability, but this study design was chosen to improve current barriers in our site’s ED. While the self-selected sample method may have introduced bias, it was used to maximize study participation and was a department-wide quality improvement initiative. The small sample size limits scalability and power, and the low survey response rate may have skewed results. People who were not motivated to complete the surveys may not be interested in performing POCUS or participating in improving the clinical ED POCUS workflow. Furthermore, the low post-intervention survey response rate and different survey sample pre- to post-intervention has few respondents with paired data. This limits within-person change analysis and limits conclusions about educational impact. Also, the lack of pre-intervention knowledge and technical skills scores limits assessment of improvement post-intervention. Surveys were adapted from previously published studies and developed by the study PI with review from expert ultrasound faculty. Survey validation, reliability testing, and item analysis were not performed in this specific hospital setting or with this study population group. Therefore, conclusions from survey and knowledge assessments are limited.

The APCs had few survey responses and low clinical POCUS use, perhaps because APCs do not attend resident education conference, where we teach POCUS once a month. Also, APCs work shifts in the clinical observation unit or fast-track ED areas, where they may not have a direct supervising attending physician to perform POCUS clinically. Extrinsic factors such as problems with ED and hospital boarding and variability in staffing models and ED personnel per day/month can affect study data results and ED workflow, which may contribute to decisions on whether to perform POCUS. The surveys were de-identified to prevent unintended work influence and bias. Thus, emergency clinicians did not receive direct feedback to improve their POCUS use unless they participated during clinical shift in-person education.

We are planning a targeted training approach for emergency physicians with the most clinical hours and fewest POCUS exams performed, conducting individualized in-person learning sessions to further improve clinical ED POCUS use and documentation. Finally, protocol dissemination at additional sites can compare or contrast POCUS barriers and facilitators and adapt the intervention for each site.

## CONCLUSION

A multifaceted point-of-care ultrasound intervention with in-person teaching, an accessible reference document, and image-based learning tool did not significantly increase mean level of clinical ED POCUS use per 100 ED patients post-intervention after adjusting for an underlying secular positive trend. Detecting incremental intervention effects in academic systems with ongoing culture and educational initiatives is difficult. Future studies can incorporate individualized interventions for infrequent POCUS users or institutional-wide initiatives with POCUS clinical champions to achieve clinical POCUS improvements.

## Supplementary Information











## Figures and Tables

**Table 1:** Characteristics of survey participants by survey response pattern in a study to assess the impact of a clinical intervention to address barriers and increase clinical point-of-care ultrasound use.

	One Survey (*n* = 42)	Both Surveys (*n* = 11)	Total (*N* = 53)
What is your level of training?			
Attending physician	22 (53.7%)	8 (72.7%)	30 (57.7%)
Resident*	16 (39.0%)	3 (27.3%)	19 (36.5%)
PGY 3+	5 (31.2%)	1 (33.3%)	6 (31.6%)
PGY 2	5 (31.2%)	2 (66.7%)	7 (36.8%)
PGY 1	6 (37.5%)	0 (0.0%)	6 (31.6%)
APC	3 (7.3%)	0 (0.0%)	3 (5.8%)
Missing	1	0	1
Completed a fellowship**	9 (22.0%)	5 (45.5%)	14 (26.9%)
Missing	1	0	1
Number of years in clinical practice working in the emergency department (including residency/fellowship)			
Mean (SD)	8.0 (8.0)	8.0 (5.7)	8.0 (7.5)
Median (Q1, Q3)	6.0 (2.0, 10.0)	7.0 (3.0, 10.0)	6.5 (2.2, 10.0)
Min, Max	0.0, 32.0	1.5, 21.0	0.0, 32.0
Number of POCUS personally performed in residency training			
> 150	23 (54.8%)	10 (90.9%)	33 (62.3%)
101–150	10 (23.8%)	0 (0.0%)	10 (18.9%)
51–100	4 (9.5%)	1 (9.1%)	5 (9.4%)
0–50	5 (11.9%)	0 (0.0%)	5 (9.4%)
Number of in-person trainings attended			
0	22 (52.4%)	5 (45.5%)	27 (50.9%)
1	16 (38.1%)	6 (54.5%)	22 (41.5%)
2	4 (9.5%)	0 (0.0%)	4 (7.5%)
Survey Observation Pattern			
Pre-only	31 (73.8%)	0 (0.0%)	31 (58.5%)
Post-only	11 (26.2%)	0 (0.0%)	11 (20.8%)
Pre and Post	0 (0.0%)	11 (100.0%)	11 (20.8%)

* Percentages associated with PGY-1, PGY-2, and PGY-3+ residents are calculated with a denominator of all residents.

** Fellowships completed: health services research, emergency medical services, sports medicine, hyperbaric and undersea medicine, geriatric emergency medicine, emergency ultrasound.

*PGY*, postgraduate year; *POCUS*, point-of-care ultrasound; *SD*, standard deviation.

**Table 2:** Interrupted time series linear regression analysis pre- and post-intervention in a study to assess the impact of a clinical intervention to address barriers and increase clinical point-of-care ultrasound use.

	Mean Level Change (95% CI)	*P* value
POCUS per 100 ED patients at week 0	3.88 (3.09 to 4.66)	< .001
Slope: Pre-training	0.03 (0.01 to 0.04)	< .001
Level change: Post-training	−0.81 (−2.43 to 0.82)	.329
Slope change: Post-training	0.00 (−0.10 to 0.10)	.978
Level change: Sustainability	−2.46 (−4.91 to −0.00)	.050
Slope change: Sustainability	0.02 (−0.08 to 0.12)	.756
Slope: Post-Training	0.03 (−0.07 to 0.13)	.540
Slope: Sustainability Period	0.04 (−0.06 to 0.15)	.385

* Training period from December 2023–January 2024 was excluded from analysis.

** Durbin Watson statistic = 1.75, Breusch-Godfrey *P* value (first lag) = .179.

**Table 3:** Post-intervention evaluation participant survey responses on clinical emergency department point-of-care ultrasound training and knowledge scores in a study to assess the impact of a clinical intervention to address barriers and increase clinical point-of-care ultrasound use.

	Total (N = 22)
Did you find the ultrasound educational/training intervention useful?	
No	4 (19.0%)
Yes	17 (81.0%)
Did you find the ultrasound intervention teaching format to be effective?	
Very effective	8 (36.4%)
Somewhat effective	6 (27.3%)
Neutral	6 (27.3%)
Somewhat ineffective	2 (9.1%)
Since the POCUS intervention (1 month ago), do you feel like your ultrasound use	
Significantly increased	3 (13.6%)
Somewhat increased	10 (45.5%)
Stayed the same	9 (40.9%)
Percentage correct knowledge questions	
Mean (SD)	88.0 (12.0)
Median (Q1, Q3)	90.9 (81.8, 100.0)
Min, Max	63.6, 100.0
Passed knowledge exam	
No (0–9)	6 (27.3%)
Correct (≥ 10)	16 (72.7%)

POCUS, point-of-care ultrasound; SD, standard deviation.
