# Supplementary material for: Impact Evaluation of a Clinical Point-of-care Ultrasound Intervention in an Academic Emergency Department
Source: West J Emerg Med. 2026 Jun 28;27(4):1023–33. doi: 10.5811/westjem.53918 (PMC13436672; doi:10.5811/westjem.53918)
Supplement: Supplementary file 4 [file wjem-27-1023-s005.docx]

### Supplemental File 5. Diagnostic and procedural Butterfly POCUS exam types and exam status by study time period

|  | Study Time Period | | | |
| --- | --- | --- | --- | --- |
|  | Pre-Intervention | Post-Intervention | Sustainability Period | Total |
| N (%) | 6708 (36.6%) | 2603 (14.2%) | 8997 (49.1%) | 18308 (100.0%) |
| Status |  |  |  |  |
| Draft | 1,869 (27.9%) | 727 (27.9%) | 3,124 (34.7%) | 5,720 (31.2%) |
| Finalized | 4,700 (70.1%) | 1,792 (68.8%) | 5,574 (62.0%) | 12,066 (65.9%) |
| Pending attestation | 139 (2.1%) | 84 (3.2%) | 299 (3.3%) | 522 (2.9%) |
| QA Review Status (Across All Scans) |  |  |  |  |
| Not reviewed | 5,931 (88.4%) | 2,199 (84.5%) | 6,125 (68.1%) | 14,255 (77.9%) |
| Reviewed | 777 (11.6%) | 404 (15.5%) | 2,872 (31.9%) | 4,053 (22.1%) |
| QA Review Status (Amongst Finalized) |  |  |  |  |
| Not reviewed | 3,923 (83.5%) | 1,388 (77.5%) | 2,702 (48.5%) | 8,013 (66.4%) |
| Reviewed | 777 (16.5%) | 404 (22.5%) | 2,872 (51.5%) | 4,053 (33.6%) |
| Diagnostic Exam Type |  |  |  |  |
| Aorta | 468 (10.3%) | 102 (6.3%) | 84 (1.7%) | 654 (5.8%) |
| Biliary | 513 (11.2%) | 142 (8.8%) | 309 (6.2%) | 964 (8.6%) |
| Bowel | 22 (0.5%) | 9 (0.6%) | 15 (0.3%) | 46 (0.4%) |
| Cardiac | 1,315 (28.8%) | 531 (32.9%) | 2,196 (43.9%) | 4,042 (36.1%) |
| Lower Extremity Venuos/DVT | 36 (0.8%) | 15 (0.9%) | 80 (1.6%) | 131 (1.2%) |
| Lung | 286 (6.3%) | 121 (7.5%) | 496 (9.9%) | 903 (8.1%) |
| Nerve Block | 3 (0.1%) | 9 (0.6%) | 17 (0.3%) | 29 (0.3%) |
| OB/GYN | 13 (0.3%) | 0 (0.0%) | 0 (0.0%) | 13 (0.1%) |
| Ocular | 51 (1.1%) | 18 (1.1%) | 114 (2.3%) | 183 (1.6%) |
| Pediatric Abdomen | 1 (0.0%) | 0 (0.0%) | 0 (0.0%) | 1 (0.0%) |
| Renal/Bladder | 598 (13.1%) | 162 (10.0%) | 293 (5.9%) | 1,053 (9.4%) |
| Soft Tissue/MSK | 121 (2.7%) | 64 (4.0%) | 223 (4.5%) | 408 (3.6%) |
| TAUS | 363 (8.0%) | 158 (9.8%) | 418 (8.3%) | 939 (8.4%) |
| Vascular | 3 (0.1%) | 3 (0.2%) | 1 (0.0%) | 7 (0.1%) |
| eFAST/FAST | 757 (16.6%) | 278 (17.2%) | 734 (14.7%) | 1,769 (15.8%) |
| xNot Specified | 14 (0.3%) | 4 (0.2%) | 27 (0.5%) | 45 (0.4%) |
| Procedural Guidance Type |  |  |  |  |
| Abscess Drainage | 10 (17.2%) | 8 (21.6%) | 32 (25.8%) | 50 (22.8%) |
| Appendix | 1 (1.7%) | 0 (0.0%) | 1 (0.8%) | 2 (0.9%) |
| Arthrocentesis | 7 (12.1%) | 4 (10.8%) | 6 (4.8%) | 17 (7.8%) |
| Foreign Body Removal | 3 (5.2%) | 0 (0.0%) | 2 (1.6%) | 5 (2.3%) |
| Paracentesis | 31 (53.4%) | 20 (54.1%) | 70 (56.5%) | 121 (55.3%) |
| Thoracentesis/Chest Tube Placement | 6 (10.3%) | 5 (13.5%) | 13 (10.5%) | 24 (11.0%) |
| *categorical variables are summarized with frequency (%) **May include pediatric exams | | | | |
