# Supplementary material for: Impact Evaluation of a Clinical Point-of-care Ultrasound Intervention in an Academic Emergency Department
Source: West J Emerg Med. 2026 Jun 28;27(4):1023–33. doi: 10.5811/westjem.53918 (PMC13436672; doi:10.5811/westjem.53918)
Supplement: Supplementary file 5 [file wjem-27-1023-s004.docx]

### Supplemental File 4. Post-Intervention Knowledge Test Results by Question
